# scHNTL: single-cell RNA-seq data clustering augmented by high-order neighbors and triplet loss

**DOI:** 10.1093/bioinformatics/btaf044

**Published:** 2025-01-29

**Authors:** Hua Meng, Chuan Qin, Zhiguo Long

**Affiliations:** School of Mathematics, Southwest Jiaotong University, Chengdu, Sichuan 611756, China; Tangshan Institute, Southwest Jiaotong University, Tangshan, Heibei 063000, China; MICCIST Key Laboratory, School of Computing and Artificial Intelligence, Southwest Jiaotong University, Chengdu, Sichuan 611756, China; Tangshan Institute, Southwest Jiaotong University, Tangshan, Heibei 063000, China; MICCIST Key Laboratory, School of Computing and Artificial Intelligence, Southwest Jiaotong University, Chengdu, Sichuan 611756, China

## Abstract

**Motivation:**

The rapid development of single-cell RNA sequencing (scRNA-seq) has significantly advanced biomedical research. Clustering analysis, crucial for scRNA-seq data, faces challenges including data sparsity, high dimensionality, and variable gene expressions. Better low-dimensional embeddings for these complex data should maintain intrinsic information while making similar data close and dissimilar data distant. However, existing methods utilizing neural networks typically focus on minimizing reconstruction loss and maintaining *similarity* in embeddings of directly related cells, but fail to consider *dissimilarity*, thus lacking separability and limiting the performance of clustering.

**Results:**

We propose a novel clustering algorithm, called scHNTL (scRNA-seq data clustering augmented by high-order neighbors and triplet loss). It first constructs an auxiliary similarity graph and uses a Graph Attentional Autoencoder to learn initial embeddings of cells. Then it identifies similar and dissimilar cells by exploring high-order structures of the similarity graph and exploits a triplet loss of contrastive learning, to improve the embeddings in preserving structural information by separating dissimilar pairs. Finally, this improvement for embedding and the target of clustering are fused in a self-optimizing clustering framework to obtain the clusters. Experimental evaluations on 16 real-world datasets demonstrate the superiority of scHNTL in clustering over the state-of-the-arts single-cell clustering algorithms.

**Availability and implementation:**

Python implementation of scHNTL is available at Figshare (https://doi.org/10.6084/m9.figshare.27001090) and Github (https://github.com/SWJTU-ML/scHNTL-code).

## 1 Introduction

The rapid development of single-cell RNA sequencing (scRNA-seq) technology has significantly advanced biomedical research (Wan *et al.* 2022). This technology allows for detailed gene expression analysis at the single-cell level (Guo *et al.* 2018).

Unsupervised clustering is crucial in scRNA-seq data analysis (Tian *et al.* 2019, Li *et al.* 2020, Cheng and Ma 2022), as it aims to identify distinct subpopulations without prior category information. However, clustering scRNA-seq data poses several unique challenges compared to conventional data. Firstly, technical limitations (e.g. dropout events) result in a high proportion of zeros, making the data extremely sparse. Secondly, the high dimensionality of scRNA-seq data complicates distance metrics, and the “curse of dimensionality” reduces the effectiveness of traditional clustering methods (Zheng *et al*. 2017). Lastly, cells of the same type can exhibit significant variability, and the smooth transitions between cell types add to the complexity of clustering (Shalek *et al*. 2013).

To perform effective clustering on scRNA-seq data, it is essential to address these challenges and develop methods capable of handling data sparsity, high dimensionality, and variability in gene expression.

In recent years, researchers have developed various clustering algorithms to address the challenges of high dimensionality and sparsity in scRNA-seq data. Traditional methods often focus on improving cell distance (Lin *et al.* 2017) or similarity measures (Kiselev *et al.* 2017). However, they treat representation learning and clustering as separate steps, limiting mutual optimization and reducing the quality of cell representation.

To integrate representation learning and clustering for scRNA-seq data, researchers proposed to combine autoencoders (Hinton and Salakhutdinov 2006) with Deep Embedded Clustering [DEC Xie *et al.* 2016)].

For example, scDeepCluster (Tian *et al.* 2019) uses an autoencoder with a loss based on a zero-inflated negative binomial distribution to capture the sparsity of scRNA-seq data and applies a clustering loss to the latent representation for joint optimization. DESC (Li *et al.* 2020) first trains an autoencoder with reconstruction loss, then initializes the DEC embedding network with the encoder, and performs clustering through iterative self-learning using the Kullback–Leibler (KL) divergence between clustering results and a target distribution. The graphical structure of data also provides valuable information for clustering. scGNN (Wang *et al.* 2021) utilizes a Graph Convolutional Network [GCN (Kipf and Welling 2017)] to incorporate connection information from the similarity graph into the autoencoder framework, leading to more precise and robust clustering results. Later, scGAC (Cheng and Ma 2022) improves scGNN by using network denoising techniques and replacing GCN with a Graph Attentional Network [GAT (Veličković *et al.* 2018)] to address noisy connections and differentiate neighbors with varying connection strengths. In addition to the use of GAT, scGCC (Tian *et al.* 2023) also makes use of data augmentation techniques to obtain positive and negative pairs for feature representation learning. scCDCG (Xu *et al.* 2024) considers cosine similarity and feature covariance of cells to construct to graphs and uses spectral loss to obtain better embeddings for clustering.

Note that existing methods only use direct connections in similarity graphs to embed connected cells closely. This would miss subtle similarities in cells and make cells from the same category be embedded further apart. Moreover, they ignore dissimilarities of cells and may embed dissimilar data closely, resulting less separable embeddings for dissimilar data.

Contrastive learning (van den Oord *et al.* 2018) is a well-known framework for enhancing data separability. Several works (Chen *et al.* 2023, Tian *et al.* 2023) in bioinformatics have adopted this framework to improve the accuracy and robustness of feature representation, e.g. Chen *et al.* (2023) exploits contrastive learning for cancer subtype identification.

In this paper, we introduce scHNTL, which novelly integrates triplet loss (Schroff *et al.* 2015) from contrastive learning to improve the separability of embeddings. scHNTL also leverages high-order neighbor information of cells to identify subtle similarities and thus to balance positive and negative examples for contrastive learning, so that cells from the same category could be embedded together while others are faraway. This approach helps the autoencoder learn a more clustering-friendly latent representation, ultimately improving clustering accuracy and robustness in identifying cell subpopulations in scRNA-seq data.

The effectiveness of scHNTL has been validated across 16 real-world scRNA-seq datasets. Compared to existing clustering methods, scHNTL shows significant advantages across multiple metrics, demonstrating its superiority in handling heterogeneous and complex scRNA-seq data.

## 2 Materials and methods

### 2.1 Architecture of scHNTL

**Figure 1.**
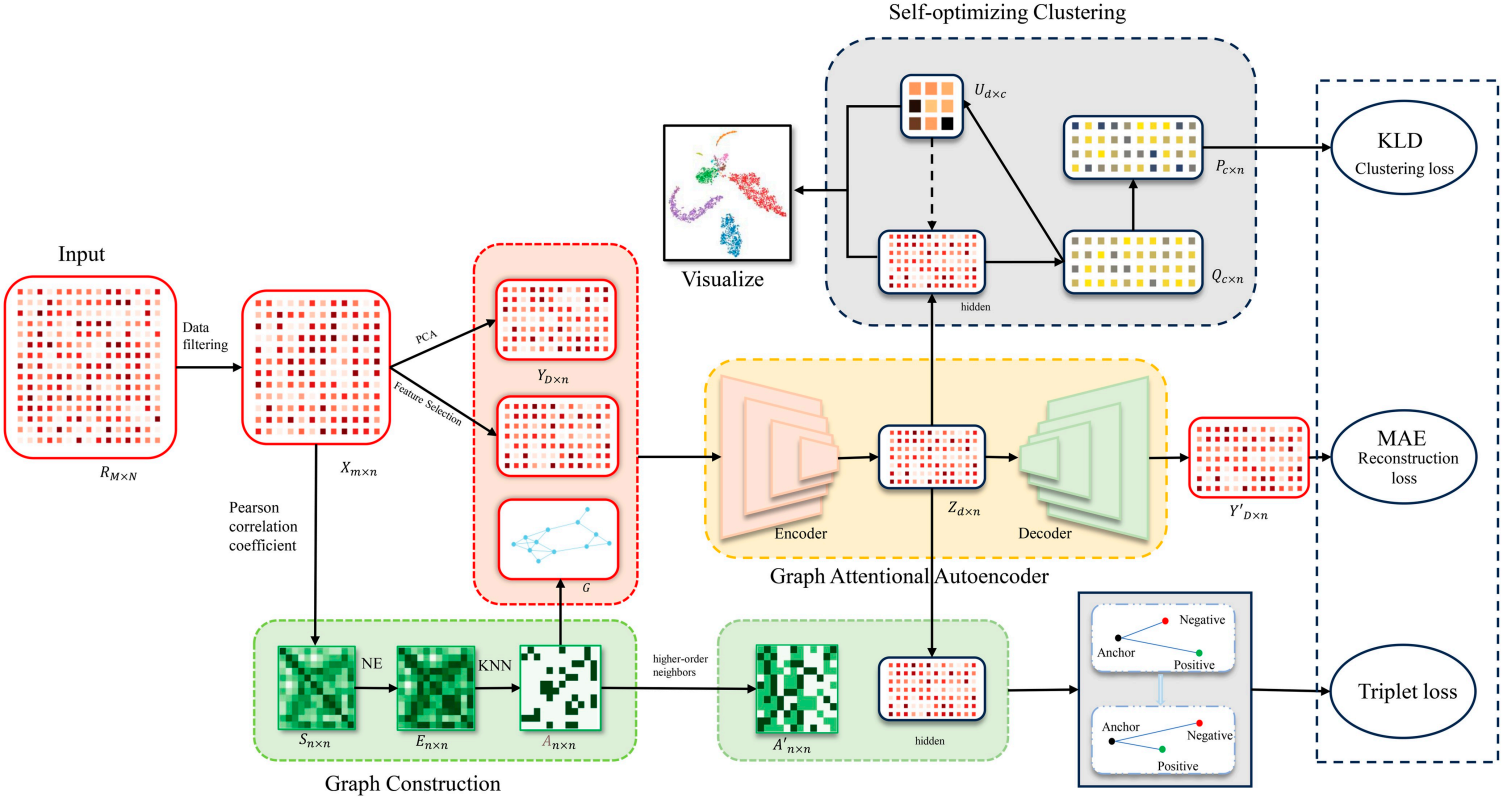
The structure of scHNTL. First, the expression matrix $R_{M\times N}$ is preprocessed and the cell graph *G* is constructed by selecting *K* most similar neighbors with the measured and denoised cell–cell similarity matrix $E_{n\times n}$. The positive and negative example mask matrix, $A_{n\times n}^{\prime }$, is derived through high-order neighbors. By dimensionality reduction, $X_{m\times n}$ is reduced to $Y_{D\times n}$. The Graph Attentional Autoencoder takes $Y_{D\times n}$ and *G* as inputs and generates a reconstructed feature matrix $Y_{D\times n}^{\prime }$, and then the reconstruction loss MAE is calculated. The self-optimizing clustering module optimizes the clustering loss of the KL divergence between the membership distribution $Q_{c\times n}$ and the target distribution $P_{c\times n}$. The triplet loss module calculates the hidden layer triplet loss using the mask $A_{n\times n}^{\prime }$. Finally, the joint loss of these three losses is used to guide model training.

The overall framework of scHNTL is shown in Fig. 1. scHNTL uses a raw gene expression matrix $R_{M\times N}$ as input, representing *M* genes and *N* cells. To facilitate further analysis, low-quality genes and cells are removed from $R_{M\times N}$ and then the resulting matrix is normalized to $X_{m\times n}$. Dimensionality reduction or feature selection is then applied to $X_{m\times n}$ to obtain a new expression matrix $Y_{D\times n}$ The following diagram illustrates the structure of scHNTL, which mainly comprises the following components:

- Graph construction: To facilitate information transfer among cells, an auxiliary cell graph *G* is constructed. To obtain the adjacency matrix $A_{n\times n}$ of *G*, the similarity matrix $S_{n\times n}$ is constructed first using Pearson correlation coefficients. A network enhancement technique [NE (Wang *et al.* 2018)] is then applied $S_{n\times n}$ to remove noisy and an enhanced matrix $E_{n\times n}$ is obtained. Finally, the K-Nearest Neighbors algorithm with $E_{n\times n}$ as the similarity matrix is used to give the adjacency matrix $A_{n\times n}$.
- Graph attentional autoencoder (Veličković *et al.* 2018): Graph Attentional Autoencoder learns low-dimensional embeddings $Z_{d\times n}$ for cells based on the input cell graph *G* and cell representations $Y_{D\times n}$. The value *d* refers to the number of neurons in the bottleneck layer of the neural network. The Mean Absolute Error (MAE) loss function is used to guide the learning process of the autoencoder.
- Self-optimizing clustering: Given the number *c* of target clusters, a membership matrix $Q_{c\times n}$ is calculated, representing the probability of each cell belonging to a specific cluster. A target distribution matrix $P_{c\times n}$, is then computed from $Q_{c\times n}$. The KL divergence between $Q_{c\times n}$ and $P_{c\times n}$ is used to adjust the cell embeddings, thereby optimizing the clustering results.
- Triplet loss: Triplet loss aims to help the model better differentiate between positive and negative examples. Based on the given cell graph *G*, a weighted adjacency matrix of the high-order neighbor graph $G^{\prime }$ is calculated, where values > 0 indicate positive examples and their weights, while the others indicate negative examples. The low-dimensional cell representations obtained from the hidden layer of GAT are used to calculate the triplet loss. This triplet loss guides the model in learning cell embeddings more suitable for clustering.

### 2.2 Details of each module

#### 2.2.1 Graph construction

To discover and learn the potential relationships between cells, it is important to share information among similar cells, and learn cell embeddings that are more suitable for clustering. Constructing a cell graph based on the similarity between cells is a common practice, since there are no inherent relationships between cells in the original scRNA-seq data.

We first determine the similarity between cells by calculating the Pearson correlation coefficient, resulting in an initial similarity matrix $S_{n\times n}$. However, due to the high dimensionality and noisy nature of scRNA-seq data, there can be considerable errors in the calculation of similarity between cells, which may affect the subsequent learning process based on similarity. Here, we use the Network Enhancement [NE (Wang *et al.* 2018)] method to denoise $S_{n\times n}$. NE can remove edges with smaller weights and enhance edges with larger weights in a similarity matrix. After applying NE to $S_{n\times n}$, we obtain a reweighted similarity matrix ${\hat{E}}_{n\times n}$, and then follow (Cheng and Ma 2022) to calculate the denoised weighted adjacency matrix $E_{n\times n}$ from ${\hat{E}}_{n\times n}$ as: $E_{ij}=S_{ij}$, if ${\hat{E}}_{ij}\geq t$, and $E_{ij}=0$ otherwise, where *t* is a predefined threshold.

If the enhanced similarity ${\hat{E}}_{ij}$ < *t*, then it is considered to be noise, and that position is set to 0. By using this method, a more accurate cell graph *G* that reflects the similarity relationships between cells can be constructed, by selecting the *K* neighbors with the largest similarities to form the adjacency matrix $A_{n\times n}$.

#### 2.2.2 High-order neighbors

After obtaining the similarity graph *G*, if we use this graph directly to distinguish positive and negative examples for triplet loss, there might not be enough positive examples but too many negative examples, making it hard to learn intrinsic neighboring relationships. This could also result in cell pairs that are actually from the same category being incorrectly assigned as negative examples, leading to suboptimal performance.

To resolve this issue, we make use of high-order neighbors: for a node (cell) in the graph, a *k*th order neighbor is a node reachable from the node via a path of length *k* and there is no path of length < *k*. The corresponding positions in the adjacency matrix for these high-order neighbors are assigned with appropriate weights $\omega_{k}$. Algorithm 1 shows the specific procedure to obtain high-order neighbors. The idea to obtain the *k*th order neighbors of cells is to first calculate the matrix $A^{k}$ indicating the cells that are connected by paths of length *k*, i.e. $A^{k}$, and then to remove those that have already been connected by paths of length smaller than *k*, i.e. $C_{k-1}\&A^{k}$. The matrix $A^{\prime }$ accumulates the weighted neighboring relationships of orders from 1 to *r*, and $C_{r}$ accumulates the zero-one neighboring information of orders from 1 to *r*. This technique increases the number of positive examples while also enabling effective differentiation of cell pairs of varying similarities.

Algorithm 1: High-Order Adjacency Matrices

**Input:** Adjacency matrix *A*, maximum order *r*, weight list ω

**Output:** Weighted high-order adjacency matrix $A^{\prime }$ and cumulative adjacency matrix $C_{r}$.

1: $A^{\prime }\leftarrow 0$▹ with the same size as *A*

2: $C_{1}\leftarrow A$

3: A←A&1▹  1 is the all-ones matrix, & is element-wise logical AND

4:  **for**  k=2 to *r* **do**

5: $A^{k}\leftarrow A^{k}\&1$

6: $A^{\prime }\leftarrow A^{\prime }+\left[A^{k}-(C_{k-1}\&A^{k})\right]*\omega_{k}$

7:$C_{k}\leftarrow A^{k}|C_{k-1}$     ▹ | is element-wise logical OR

8: **end for**

9: **return**  $A^{\prime },C_{r}$

*Difference with existing graph-based methods*: Most existing methods (Cheng and Ma 2022, Abadi *et al.* 2023) use only direct neighbors to construct cell graphs, which limits the number of positive examples and hinders effective learning of subtle cell similarities. Our approach leverages high-order neighbors and assigns appropriate weights to capture varying connection strengths, which helps to identify similar cells in the complex biological data. High-order neighbors also enrich positive examples for a more balanced triplet loss, and thus improve the embeddings of cells for more effective clustering.

#### 2.2.3 Triplet loss

For an autoencoder, because it only tries to minimize the reconstruction loss, composing any invertible transformation *h* to the encoder *f* and the corresponding inverse transformation $h^{-1}$ to the decoder *g* will give another encoder h°f and decoder $g{}^{\circ}h^{-1}$, which provide different latent representation but the same reconstruction performance as the original *f* and *g*. Therefore, a good model for clustering should be equipped with components that ensures the learned low-dimensional representation of cells is more suitable for clustering. Triplet loss is a loss function commonly used in contrastive learning for metric and similarity learning. The goal is to make representations of data from the same category closer, while those from different categories further away. Here, *category* refers to supervised learning labels or clusters automatically identified during unsupervised learning. Triplet loss achieves this by considering three data samples at once: an anchor *a*, a positive example *p*, and a negative example *n*. The general triplet loss can be formally written as:

(1) max{d(a,p)−d(a,n)+margin,0}

Here, *d* represents the distance or dissimilarity between the positive (negative) example and the anchor.

In fact, anchors are representative points of data to reflect the overall distribution, and a positive example are considered to be highly possible to belong to the same category of the anchor, while a negative example is likely to belong to a different category. Minimizing the above loss in Equation (1) will ensure the difference between the positive and negative examples is large enough, i.e. the distance between the anchor and the positive example is at least one margin smaller than that for the negative example. Note that if this is not satisfied, then the loss value will be positive; otherwise, it is zero. This encourages the model to bring similar items closer and push dissimilar items further apart during training.

In this paper, instead of the direct adjacency information *A*, weighted high-order neighbor information $A^{\prime }$ is used to determine positive and negative examples: given an anchor cell, its direct and higher-order neighbors are all treated as positive examples, and the rest are negative examples. The triplet loss $L_{\text{triplet}}$ used in the proposed model is then formulated as:

(2) $$\max\{\frac{1}{n_{+}}\sum_{i,j}A_{ij}^{\prime }\times S_{ij}^{\prime }-\frac{1}{n_{-}}\sum_{i,j}B_{ij}\times S_{ij}^{\prime }+\mathrm{margin},0\},$$

where $S^{\prime }$ is the similarity matrix obtained by calculating the Pearson correlation coefficients of the low-dimensional representation $Z_{d\times n}$, $B=1-C_{k}$ is the all-ones matrix and $n_{+}$ and $n_{-}$ are the total numbers of positive examples and negative examples, respectively.

*Difference with existing clustering methods*: Existing clustering methods using autoencoders often focus solely on minimizing reconstruction loss (Li *et al.* 2020), or on making the embeddings of the cells that are likely from the same category more compact (Cheng and Ma 2022). This either leads to that embeddings are not suitable for clustering, since representation learning and clustering are separate, or the separability of dissimilar cells is not enough, since there is no requirement for that when optimizing the embeddings. In contrast, our approach integrates high-order neighbors and triplet loss into the self-optimizing clustering process, which can jointly optimize representation learning and clustering, and improve the distinguishability of similar and dissimilar cells by requiring that similar cells should be more close than dissimilar cells by a given margin.

#### 2.2.4 Graph attentional autoencoder

Based on the Graph Attention Network (Veličković *et al.* 2018), we make use of a Graph Attentional Autoencoder to embed the input cell expression into a low-dimensional space. This autoencoder consists of two graph attentional layers in the encoder and symmetric layers in the decoder.

For a given cell graph, the graph attentional layer learns cell latent representations by accumulating weighted neighboring expressions. This would result in similar representations being learnt for neighboring cells. Specifically, a graph attentional layer can be formulated as $h_{i}^{\prime }=\sigma (\sum_{j\in N_{i}}\alpha_{ij}Wh_{j})$, where $h_{i}^{\prime }$ is the new low-dimensional latent representation for cell *i*, $N_{i}$ is the set of neighbors for cell *i* in the cell graph *G*, $h_{j}$ is the original expression for cell *j*, *W* is a learnable transformation matrix, σ is a nonlinear activation function (ELU function in this paper), and $\alpha_{ij}$ is the weight coefficient representing the importance of cell *j* to cell *i*.

Following (Cheng and Ma 2022), we explicitly integrate similarity information $e_{ij}$ when calculating the attentional coefficients $\alpha_{ij}$. This similarity information is derived by applying a Gaussian kernel to the weighted distance between two cells:

(3) $$e_{ij}=\text{exp}\;\left(-{\left|a_{i}^{T}Wh_{i}-a_{j}^{T}Wh_{j}\right|}^{2}\right)$$

where $a_{i}$ is a learnable weight vectors for cell *i*. Then the attentional coefficients are obtained by normalizing $e_{ij}$ using the softmax function to enable comparison across different cells:

(4) $$\alpha_{ij}={\text{softmax}}_{j}(e_{ij})=\frac{\;\text{exp}\;\left(e_{ij}\right)}{\sum_{k\in N_{i}}\;\text{exp}\;\left(e_{ik}\right)}$$

We also use a multi-head attention mechanism to stabilize the learning process. Using *T* independent attentional modules to learn representations collectively can be expressed as:

(5) $$h_{i}^{\prime }={⊕}_{t=1}^{M}\sigma (\sum_{j\in N_{i}}\alpha_{ij}^{t}W^{t}h_{j})$$

In our implementation, the processed cell expression matrix $Y_{D\times n}$ and the adjacency matrix $A_{n\times n}$ of cell graph *G* are fed into the Graph Attentional Autoencoder, where $Y_{D\times n}$ provides gene-level expression information and $A_{n\times n}$ provides cell-level neighboring information. To guide the learning process, the Mean Absolute Error (MAE) is calculated between the reconstructed feature matrix $Y_{D\times n}^{\prime }$ by the autoencoder and the original expression matrix $Y_{D\times n}$ as the reconstruction loss:

(6) $$L_{r}=\sum_{i=1}^{D}\sum_{j=1}^{n}\left|Y_{ij}-Y_{ij}^{\prime }\right|$$

#### 2.2.5 Self-optimizing clustering

After training the Graph Attentional Autoencoder, we can extract the cell embeddings $Z_{d\times n}$ from the hidden layer. These embeddings are derived from both cell–cell relationships and gene expression features. With the new representation of cells, we can perform K-means clustering to get an initial clustering result.

However, at this stage, the clustering module and the feature learning module do not interact with each other, leading to potential inaccuracies in the clustering results. This is because the embeddings from the autoencoder might not be optimized for clustering. To address this issue, a self-optimizing clustering approach is adopted to ensure that the feature learning and clustering modules benefit from each other during the training process, ultimately achieving better clustering results. Based on the Deep Embedded Clustering [DEC (Xie *et al.* 2016)] method, we first initialize the cluster centers with K-means and measure the probability of a cell belonging to a center using the Student’s *t* distribution. We denote by *Q* the matrix representing this probability, where $q_{ij}$ is normalized as follows.

(7) $$q_{ij}=\frac{{\left(1+{\left\|z_{i}-u_{j}\right\|}^{2}\right)}^{-1}}{\sum_{k=1}^{c}{\left(1+{\left\|z_{i}-u_{k}\right\|}^{2}\right)}^{-1}}$$

Here, $z_{i}$ is the low-dimensional embedding of cell *i* in the matrix $Z_{d\times n}$ learned by the autoencoder. The cluster center $u_{j}$ represents the centroid for each cluster from K-means, and *c* denotes the number of cell categories. Using the membership matrix *Q* we can derive an additional relationship matrix *P* as

(8) $$p_{ij}=\frac{q_{ij}^{2}/\sum_{i=1}^{n}q_{ij}}{\sum_{k=1}^{c}\left(q_{ik}^{2}/\sum_{i=1}^{n}q_{ik}\right)}$$

Here, by squaring $q_{ij}$, *P* tries to improve cluster purity, as smaller values will become much smaller after being squared, and the normalization can balance the effect of cluster size and density.

Using the matrices *P* and *Q*, we can update the cluster centers through the weighted average of the cell expressions:

(9) $$u_{j}=\frac{\sum_{l_{i}=j}q_{ij}z_{i}}{\sum_{l_{i}=j}q_{ij}}$$

where $l_{i}=\arg\max_{j}q_{ij}$ represents the clustering label of cell *i* in the current iteration.

The self-optimizing clustering is achieved by updating *Q* distribution to be similar to the adjusted *P* distribution using the KL divergence between $P_{c\times n}$ and $Q_{c\times n}$ as the clustering loss:

(10) $$L_{c}=\sum_{i=1}^{c}\sum_{j=1}^{n}p_{ij}\;\text{log}\;\frac{p_{ij}}{q_{ij}}$$

#### 2.2.6 Joint loss

The joint loss function combines the loss from the Graph Attentional Network (GAT), the clustering loss, and the triplet loss calculated from the hidden layer based on high-order neighbors:

(11) $$L=L_{r}+\alpha L_{c}+\beta L_{\text{triplet}}$$

where α and β are hyperparameters used to balance the contributions of the three loss functions.

This comprehensive loss function aims to simultaneously optimize multiple aspects of model performance, including effective embedding of the original data features in the hidden layer, clustering quality, and preservation of high-order adjacency relationships in the embedding space.

Furthermore, we use the Silhouette score on the hidden layer to guide the early stopping of the training: $s_{i}=\frac{b_{i}-a_{i}}{\max(a_{i},b_{i})}$.

### 2.3 Datasets and pre-processing

#### 2.3.1 Datasets

We evaluate the performance of the scHNTL algorithm on a variety of real-world datasets covering samples of different biological species, various tissue types, and multiple sequencing platforms. The datasets (Table 1) include small size but high dimensional ones, such as Biase, which only contains 49 cells and 3 cell types, with each cell having 21 489 genes; large-scale ones with a large number of cells, such as Sun3, which contains 8197 cells, covering 7 cell types, with each cell having 1000 genes. The cell type annotations from the original publications were used as the ground truth of *data categories*.

#### 2.3.2 Pre-processing

Raw scRNA-seq data typically exhibit high dimensionality, sparsity, and noise, requiring quality control steps for filtration. In the initial phase of the pre-processing workflow, thresholds are set to ensure that the gene expression of each cell falls within a reasonable range, and thus noisy data are properly identified and handled. The upper and lower bounds are determined by adding and subtracting the interquartile range to and from the 75th and 25th percentiles, respectively. Cells falling outside this range are considered low-quality and removed. Additionally, genes expressed in only a few cells (≤3) are also removed. After the above steps, the read counts in the original expressions are normalized for further analysis as in Cheng and Ma (2022). To enhance efficiency in subsequent analysis and to resolve the sparsity issue of features, dimensionality reduction is performed before feeding the data into the Graph Attentional Autoencoder. To ensure the input dimensionality is *D*, Principal Component Analysis (PCA) is used to reduce the dimensionality of data, but for smaller datasets (where the number of dimensions exceeds the number of samples), the *D* most variable genes are selected instead. After dimensionality reduction, following the literature, e.g. Cheng and Ma (2022), the data are standardized using z-score normalization to balance the influence of features.

### 2.4 Benchmark settings

#### 2.4.1 Parameter settings

We implemented scHNTL using TensorFlow 1.12 with Python 3.6. It consists of 4 heads of graph attentional autoencoder, each of which contains 2 layers and each layer is of size (512, 256, 64). The learning rate for training the autoencoder is 0.0002 and it is first trained with early stopping enabled for 100 epochs with the MAE loss function. Subsequently, the whole model with the joint loss is trained for an additional 2000 epochs with a learning rate of 0.0005 and early stopping enabled. The number of nearest neighbors *K* used for constructing the cell graph is determined by $K=\text{round}(N_{\text{cells}}/(10\times N_{\text{clusters}}))$ and we set *K* to 20 if K>20, and to 6 if K<6. In the calculation of triplet loss, the hyperparameter margin was set to 0.2, and the hyperparameters α and β, used to balance the three parts of the total loss, were both set to 1. The detailed information about decision of parameters margin and neighbor order refers to Supplementary Tables S3–S6. All experiments were conducted on a computer with an Intel W-2233 CPU and 32 GB memory.

#### 2.4.2 Comparison methods

We used six recent scRNA-seq clustering methods, i.e. CIDR (Lin *et al.* 2017), DESC (Li *et al.* 2020), SC3 (Kiselev *et al.* 2017), scDeepCluster (Tian *et al.* 2019), scGAC (Cheng and Ma 2022), and SCEA (Abadi *et al.* 2023) and 2 baseline clustering methods, i.e. t-SNE (van der Maaten and Hinton 2008) and PCA (Jolliffe 2011). All methods were configured with their default values or according to their respective guidelines. For CIDR and scDeepCluster, we provide the original data for subsequent preprocessing and clustering steps. For SC3, we use either the original or log-transformed data, depending on its specifications. We use DESC with normalization, log-transformation, gene selection, and scaling according to its guidelines, and the results generated at a “resolution” of 0.8 are taken as the final clustering results. For scGAC, the parameter settings used are the same as for the proposed scHNTL. Here, we also used commonly used PCA+K-means and t-SNE+K-means methods as baseline methods to verify whether simple dimensionality reduction followed by clustering is always applicable to scRNA data.

**Table 1.** Datasets and their characteristics.

Datasets	Description	Cells	Genes	Cell types	Platform	Reference
Adam	Kidney	3406	17 310	8	Drop-seq	Adam *et al.* (2017)
Baron1	Human pancreas	1918	14 709	14	inDrop	Baron *et al.* (2016)
Baron2	Human pancreas	1721	14 881	14	inDrop	Baron *et al.* (2016)
Baron3	Human pancreas	3487	15 167	14	inDrop	Baron *et al.* (2016)
Baron4	Human pancreas	1289	14 439	14	inDrop	Baron *et al.* (2016)
Baron Mouse	Mouse pancreas	1883	14 461	13	inDrop	Baron *et al.* (2016)
Biase	Mouse embryos	49	21 489	3	Smart-Seq	Biase *et al.* (2014)
Björklund	Human tonsil ILCs	647	26 087	4	Smart-Seq2	Björklund *et al.* (2016)
Brown2	Human melanoma cells	8605	16 736	7	Chromium	Brown et al. (2019)
Klein	Mouse embryonic stem cells	2717	24 021	4	inDrop	Klein *et al.* (2015)
PBMC^a^	Human peripheral blood mononuclear cells	5356	14 219	4	Smart-Seq2	
Qs_Diaphragm	Diaphragm	869	15 027	5	Smart-Seq2	Schaum *et al.* (2018)
Qx_Bladder	Bladder	2485	15 357	6	Smart-Seq2	Schaum *et al.* (2018)
Romanov	Mouse hypothalamus	2089	18 946	7	Drop-Seq	Romanov *et al.* (2017)
Sun2	Human skin cells	4717	999	8	Chromium	Sun *et al.* (2019)
Sun3	Human peripheral blood mononuclear cells	8197	1000	7	Chromium	Sun *et al.* (2019)

a Dataset PBMC is obtained from https://support.10xgenomics.com/single-cell-gene-expression/datasets/1.1.0/pbmc6k.

#### 2.4.3 Cell embedding analysis

For visualization purposes, we will use t-distributed stochastic neighbor embedding [t-SNE (van der Maaten and Hinton 2008)] to reduce the dimensionality to two, and then the points in the 2D plane will be colored based on their annotated cell types.

#### 2.4.4 Evaluation metrics

To evaluate the clustering results of scHNTL and baseline methods, we used two popular metrics to assess the clustering results of the algorithms: the Adjusted Rand Index (ARI) and the Normalized Mutual Information (NMI). These two metrics are used to quantify the consistency and information-sharing level between the clustering results and the ground-truth category labels. The detailed information of ARI and NMI can be found in Supplementary Section S1 of the Supplementary Material.

## 3 Results

### 3.1 scHNTL achieves excellent clustering performance and outperforms existing methods

**Figure 2.**
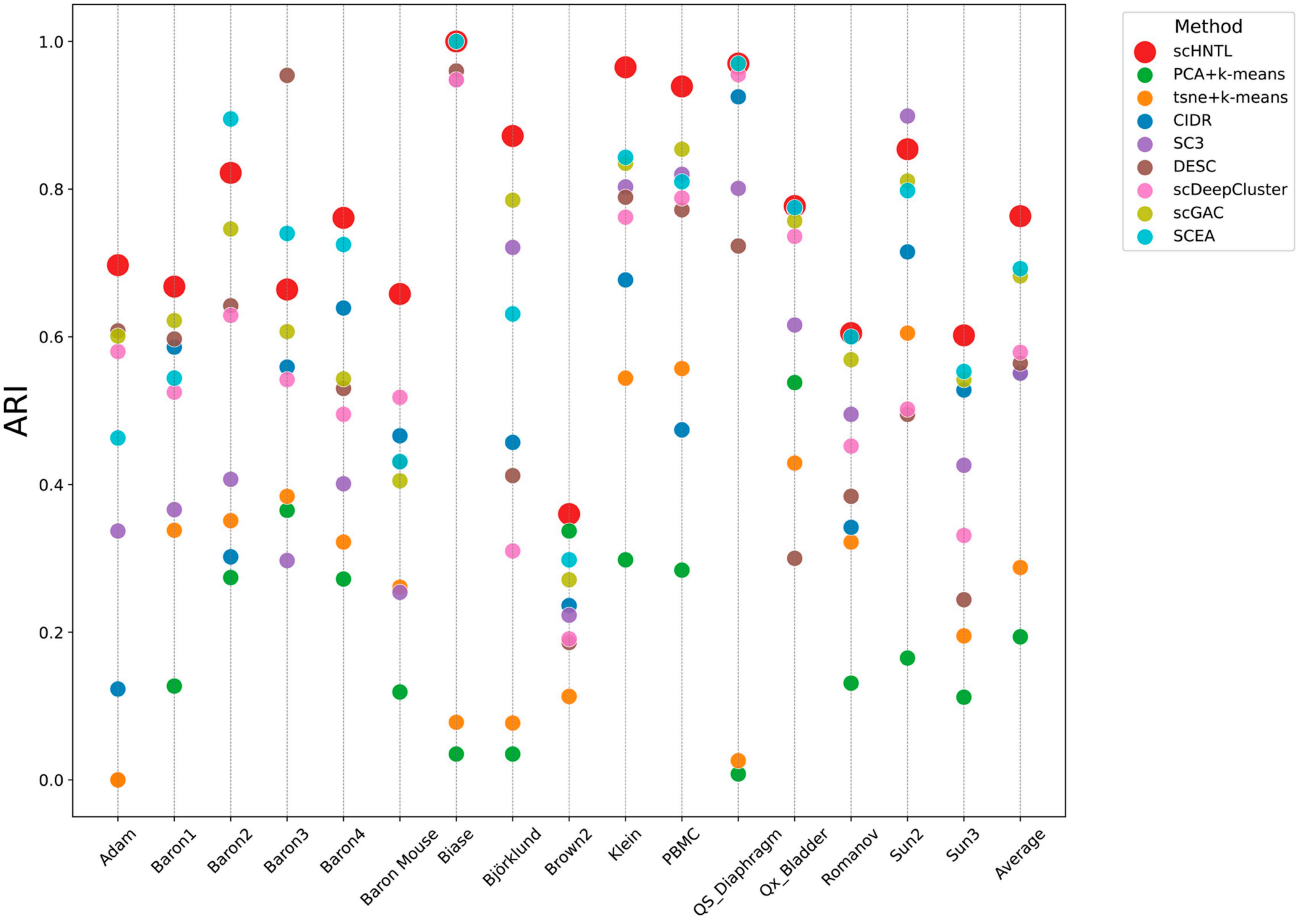
The ARIs of scHNTL and baseline methods on different datasets. Each point represents the performance of a method on a dataset, where height indicates the value of ARI. More detail information can be found in Supplementary Table S1.

We applied scHNTL to 16 real-world datasets and compared its performance with several other commonly used methods. As illustrated in the Fig. 2 and Supplementary Fig. S1, scHNTL generally demonstrated the best performance in terms of ARI and NMI scores. Out of the 16 datasets, scHNTL achieved the highest ARI score in 13 and the highest NMI score in 9. Additionally, for both ARI and NMI, scHNTL had the highest average scores across all datasets.

### 3.2 scHNTL learns clustering-friendly embedding of cells

**Figure 3.**
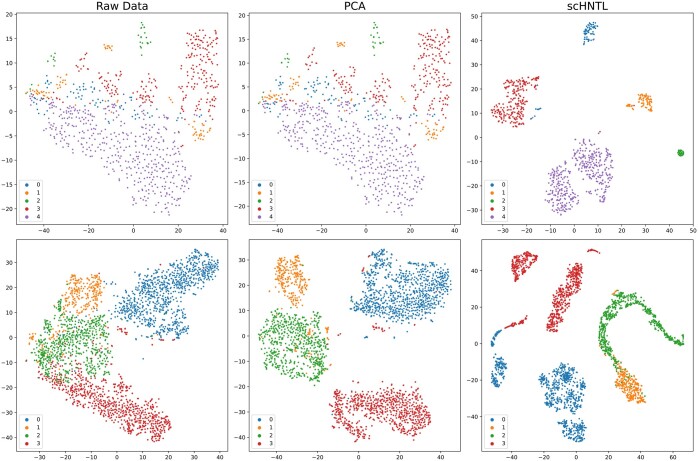
Visualization of datasets Diaphragm and Klein. The points are visualized in 2D with t-SNE and colored using the ground-truth labels.

In Fig. 3, the first, second, and third columns show the visualization of the raw data, the model input (after PCA), and the model output embeddings, respectively. From the visualizations, it can be observed that in the raw data, some boundaries are fuzzy and prone to confusion. After a simple PCA dimensionality reduction, although some data points are separated, the overall data distribution remains sparse and lacks clear clustering patterns. On the other hand, in the embeddings given by the proposed model, the boundaries of categories become clearer, and cells of the same category are more condensed. This illustrates that the proposed components can indeed improve the quality of embeddings for clustering.

### 3.3 scHNTL can find and correct erroneously clustered samples

**Figure 4.**
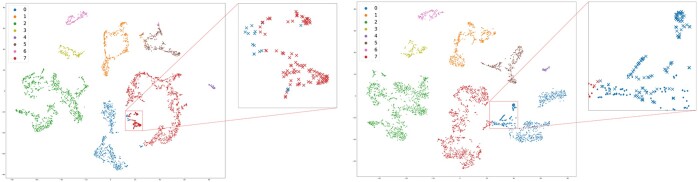
Visualization of the embeddings of dataset Sun2 *before* (left) and *after* (right) training with the joint loss. The points are visualized in 2D with t-SNE and colored using the predicted labels from the model. Before training, all the points in the rectangular box should belong to category 0, but most of the points have been clustered to the wrong category 7 when performing K-means on the embeddings. After training, the same set of points (marked with “×”) are mostly correctly clustered.

In Fig. 4, for dataset Sun2, when the embeddings *before* further training with the joint loss are used, it is clear that some cells are clustered into the wrong category because they are embedded closely to cells from another category. After the training with the joint loss, the majority of these cells are embedded correctly and thus clustered into the correct category. This demonstrates the effectiveness of scHNTL for recognizing subtle similarities of cells and enhancing separability.

### 3.4 Ablation studies

#### 3.4.1 Self-optimizing clustering module promotes clustering

One of the core components of scHNTL is the self-optimizing clustering module, responsible for providing the final clustering results. This module enhances the clustering performance by optimizing the cluster centers and reassigning membership scores. At the same time, it interacts with the autoencoder, further facilitating the process for embedding learning.

**Figure 5.**
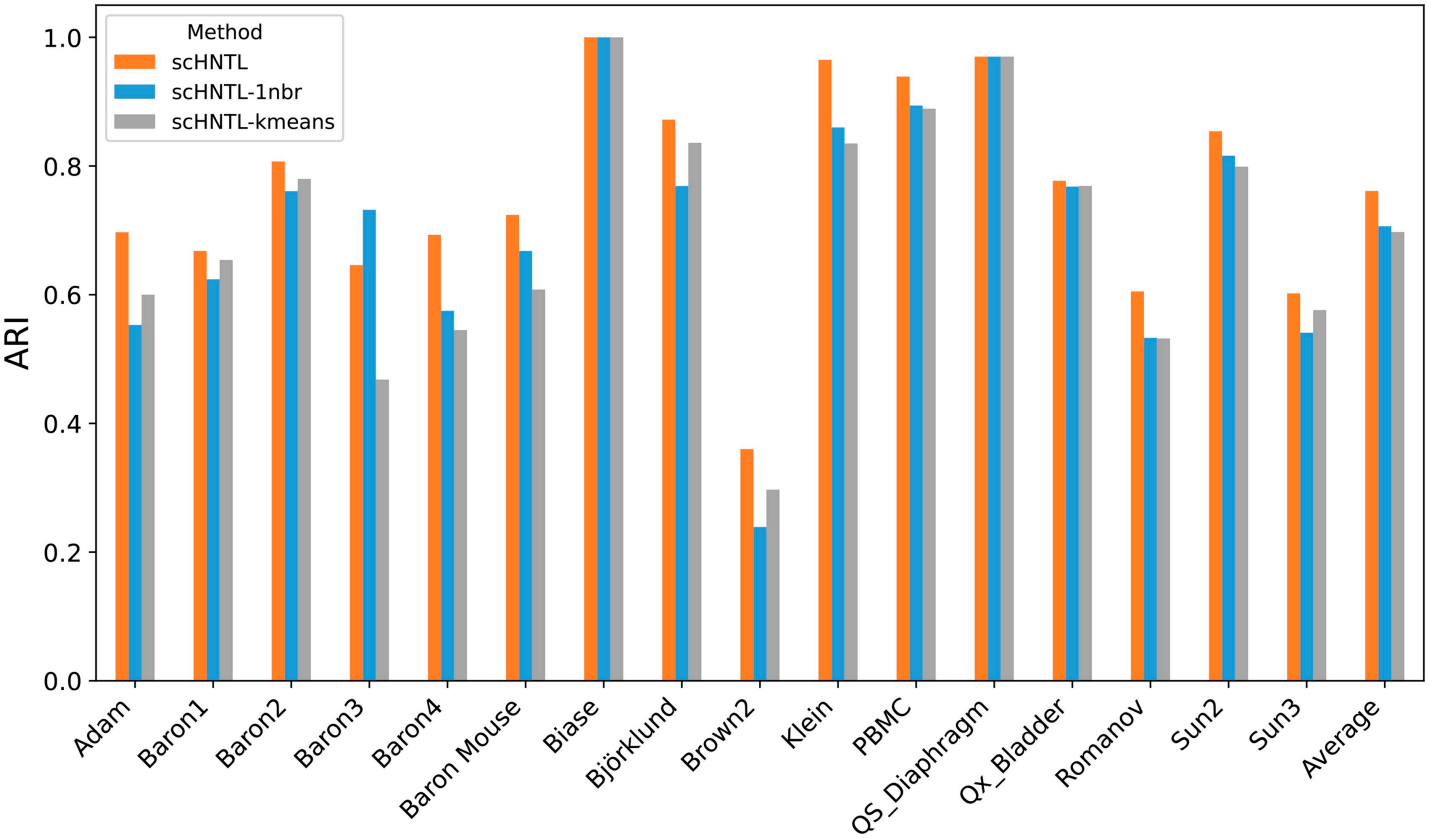
ARI scores of ablation studies, where scHNTL gets the highest ARI score on 15 out of 16 datasets.

To assess the effectiveness of this module in improving clustering results, Fig. 5 shows a comparison of clustering results with and without the self-optimizing clustering module. The results without this module (labelled as scHNTL-kmeans) were obtained by applying K-means to the pre-trained embeddings. Compared to scHNTL-kmeans, the introduction of this module in scHNTL achieved higher ARI scores on almost all datasets, confirming the effectiveness of the self-optimizing clustering module.

#### 3.4.2 High-order neighbor better captures the relationship of cells

In scHNTL, we adopted a high-order neighbor technique to compute the triplet loss, guiding the embedding learning process of the model. This high-order neighbor technique can reveal deeper adjacency relationships of cells, and resolves the problem of insufficient positive examples when using only direct neighbors.

Figure 5 also shows the comparison results between the methods relying on the original similarity graph connections (scHNTL-1nbr) and the high-order neighbors (scHNTL) to distinguish positive and negative examples. Across all datasets, the scHNTL method achieved higher scores, indicating that high-order neighbors can significantly improve the performance.

**Figure 6.**
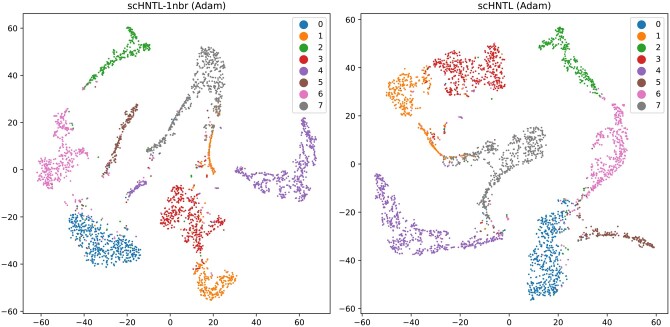
Visualize of scHNTL and scHNTL-1nbr on dataset Adam. Note that both the first and fourth categories are divided into two far-away parts by scHNTL-1nbr, while scHNTL retains them as a whole, resulting in a better performance.

Graphical analysis in Fig. 6 depicts the final representation of the Adam dataset learned by scHNTL-1nbr (left) and scHNTL (right). A clear observation is that when only using first-order neighbors, the model is hard to capture relationships among the limited number of positive examples, causing cells from the same category (category 1 and 4) to be erroneously separated, thereby reducing clustering accuracy. When high-order neighbors are introduced, subtle similarities are correctly identified and the problems no longer exist.

### 3.5 Computational cost

**Figure 7.**
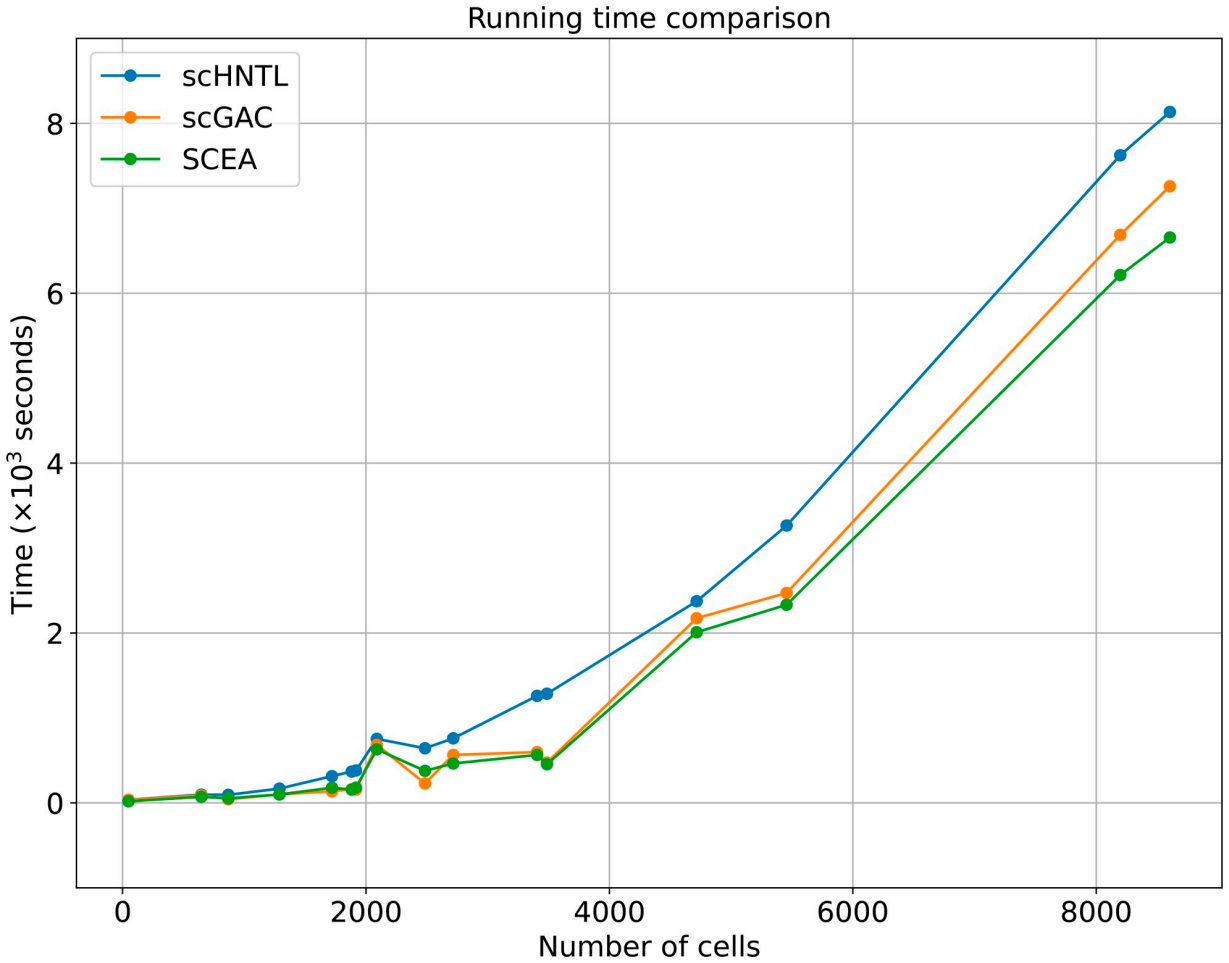
Running time comparison of scHNTL with scGAC and SCEA.

The total number of network parameters in scHNTL is approximately 1.36 million, which can be counted as a relatively small model in the current deep learning field. The worst-case time complexity of calculating the loss in one iteration is $O(n^{2})$. In Fig. 7, we also compared the running time of scHNTL with scGAC and SCEA (the others either are not deep clustering methods or did not provide code), and found that scHNTL is slightly slower than scGAC and SCEA, which is acceptable considering the significant improvement over these two methods. This level of complexity allows our model to effectively learn from scRNA-seq data while maintaining scalability.

## 4 Conclusion

In this article, we present a novel clustering method scHNTL for scRNA-seq data. To obtain more suitable cell embeddings for clustering, we introduced a triplet loss function based on high-order neighbors to better capture cell similarities and dissimilarities. By constructing a high-order cell–cell relationship graph, scHNTL better reflects the intrinsic similarities of cells, and by utilizing the triplet loss, scHNTL improves the separation for cells from different categories.

Validation on 16 real-world scRNA-seq datasets has shown that scHNTL offers significant advantages over existing clustering methods, including higher clustering accuracy and improved recognition of complex biological variations. This can benefit several downstream biological tasks (Villani *et al.* 2017, Guo *et al.* 2018), including to identify distinct cell subpopulations, and to support the discovery of novel or rare cell types by human experts.

Future improvements to scHNTL may include optimizing the construction of high-order neighbor graphs by determining the appropriate range of neighbors and assigning appropriate weights based on distance. Additionally, developing an adaptive approach to determine the margin parameter in triplet loss will provide greater flexibility, allowing the model to adapt better to various datasets and improve clustering performance. Improving the scalability is also a meaningful direction.

## Supplementary Material

btaf044_Supplementary_Data

## Data Availability

The Python implementation of scHNTL is available at Figshare (https://doi.org/10.6084/m9.figshare.27001090) and Github (https://github.com/SWJTU-ML/scHNTL-code). All datasets tested in this manuscript can be accessed through the following accession numbers or websites: Biase (GSE57249); Brown2 (GSE137710); Klein (GSE65525); Romanov (GSE74672); Brown2 (GSE137710); Sun.2 and Sun.3 [GSE128066 for original data (Processed data at https://github.com/CHPGenetics/BAMMSC.)]; Björklund (GSE70580); Baron series (https://github.com/LiShenghao813/AttentionAE-sc/tree/main/Data); Qs_Diaphragm, Qx_Bladder, and Adam (https://figshare.com/articles/software/scBGEDA/19657911); PBMC (https://support.10xgenomics.com/single-cell-gene-expression/datasets/1.1.0/pbmc6k).
