# Supplementary material for: scHNTL: single-cell RNA-seq data clustering augmented by high-order neighbors and triplet loss
Source: Bioinformatics. 2025 Jan 29;41(2):btaf044. doi: 10.1093/bioinformatics/btaf044 (PMC11878765; doi:10.1093/bioinformatics/btaf044)
Supplement: btaf044_Supplementary_Data [file btaf044_supplementary_data.zip › 0531e_supplementary materials.pdf]

Supplementary Material — "scHNTL: single-cell RNA-seq data clustering augmented by high-order neighbors and triplet loss"

Hua Meng[1], Chuan Qin[*2], and Zhiguo Long[2]

[1]School of Mathematics, Southwest Jiaotong University, No. 999, Xi'an Road, Pidu District, 611756, Sichuan, China
[2]School of Computing and Artificial Intelligence, Southwest Jiaotong University, No. 999, Xi'an Road, Pidu District, 611756, Sichuan, China

[*]Corresponding author: 2023212479@my.swjtu.edu.cn

1 Evaluation metrics

To evaluate the clustering results of scHNTL and baseline methods, we used two popular metrics to assess the clustering results of the algorithms: the Adjusted Rand Index (ARI [1]) and the Normalized Mutual Information (NMI [2]). These two metrics are used to quantify the consistency and information-sharing level between the clustering results and the ground-truth category labels. ARI is a statistical metric designed to measure the similarity between two different clusters or partitions. It corrects the original Rand Index to include the expected similarity when choosing clusters randomly, providing a more accurate measure of similarity. The specific formula for calculating ARI is as follows:

$$\text{ARI} = \frac{\sum_i \sum_j \binom{n_{ij}}{2} - \left[\sum_i \binom{a_i}{2} \sum_j \binom{b_j}{2}\right] / \binom{n}{2}}{\frac{1}{2}\left[\sum_i \binom{a_i}{2} + \sum_j \binom{b_j}{2}\right] - \left[\sum_i \binom{a_i}{2} \sum_j \binom{b_j}{2}\right] / \binom{n}{2}} \tag{1}$$

NMI is a metric derived from information theory to evaluate the level of association between two variables. It quantifies the amount of shared information between the clustering results and the ground-truth labels by normalizing the mutual information. The calculation of NMI involves the entropy of the clusters and the ground-truth labels, along with their joint entropy. The formula for calculating NMI is as follows:

$$\text{NMI} = \frac{2\,MI(U,V)}{H(U) + H(V)} \tag{2}$$

where U is the clustering result, V is the ground truth, $MI(U,V)$ represents the mutual information between U and V, and H is the entropy function.

2 Supplementary Figures

In addition to the ones in the paper, here we provide more figures to illustrate the performance of scHNTL in the experiments.

Supplementary Figure S1 shows the NMI scores in addition to the ARI scores in the paper. Out of the 16 datasets, scHNTL achieved the highest ARI score in 13 and the highest NMI score in 9. Additionally, for both ARI and NMI, scHNTL had the highest average scores across all datasets.

Supplementary Figures S2 and S3 show the changes of the ARI and NMI scores when varying the parameter margin. For most datasets, margin $= 0.2$ yields the best scores.

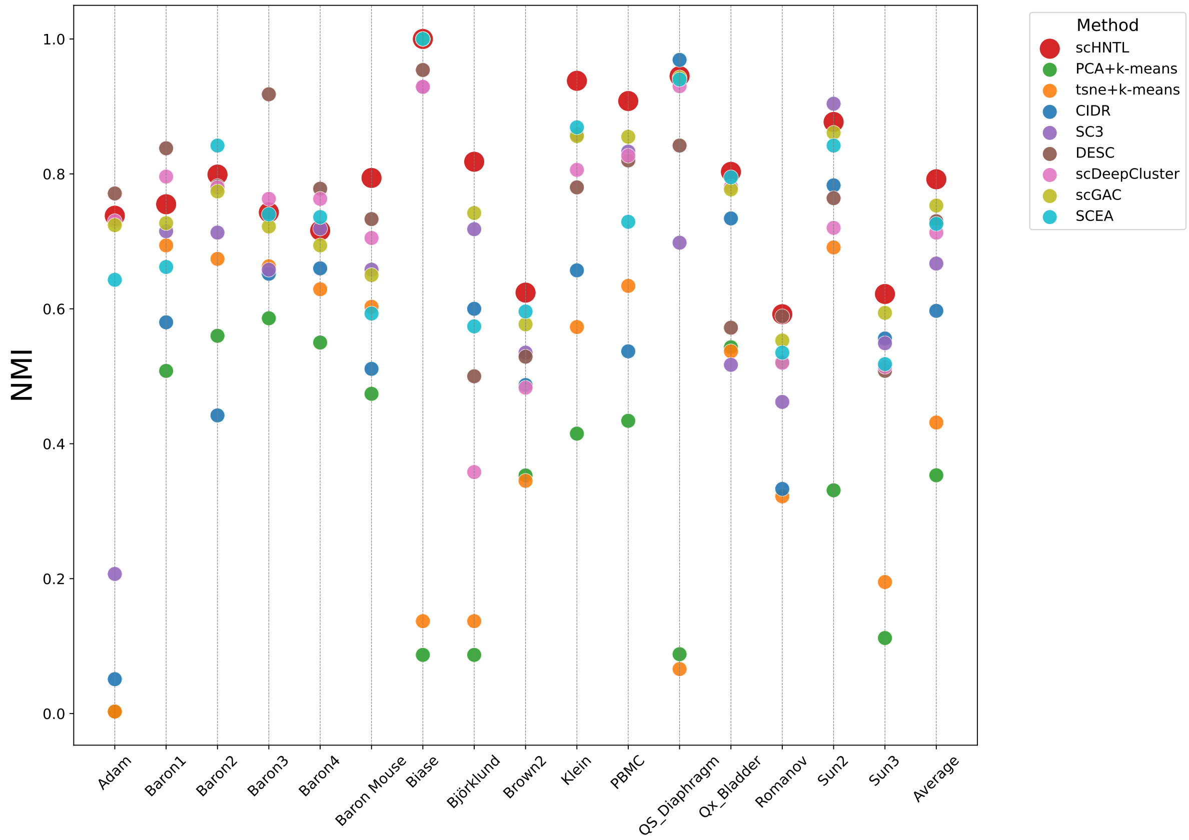

Supplementary Figure S1: The NMIs of scHNTL and baseline methods on different datasets. Each point represents the performance of a method on a dataset, where height indicates the value of NMI.

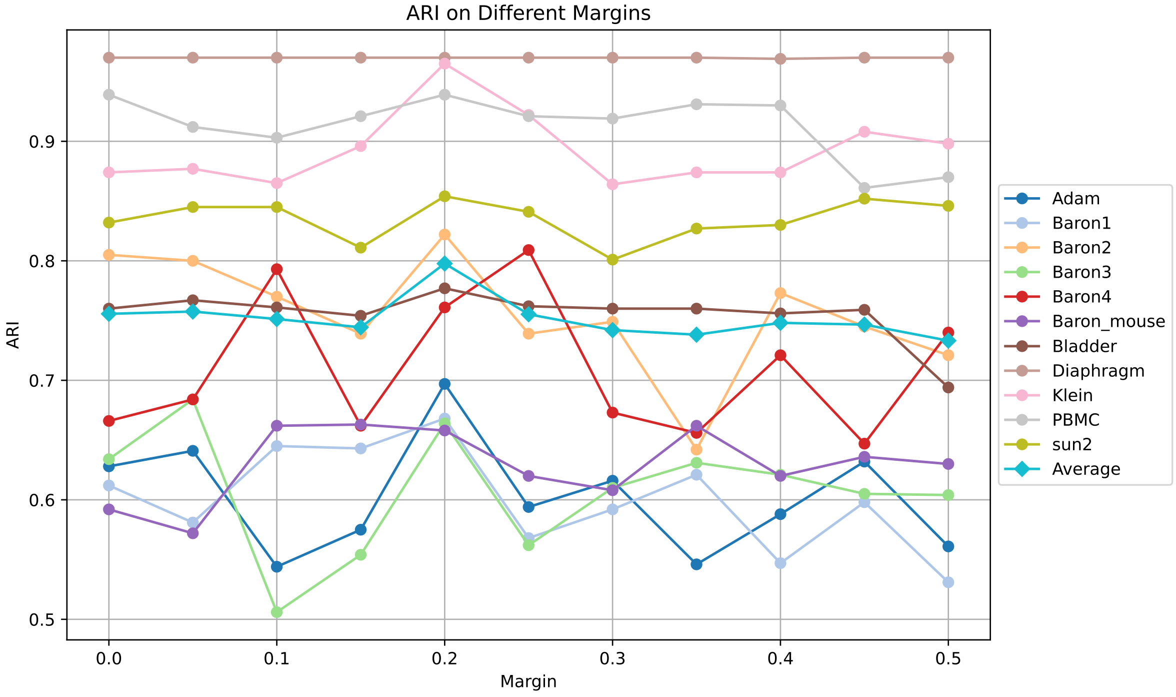

Supplementary Figure S2: The ARIs of scHNTL on different margin.

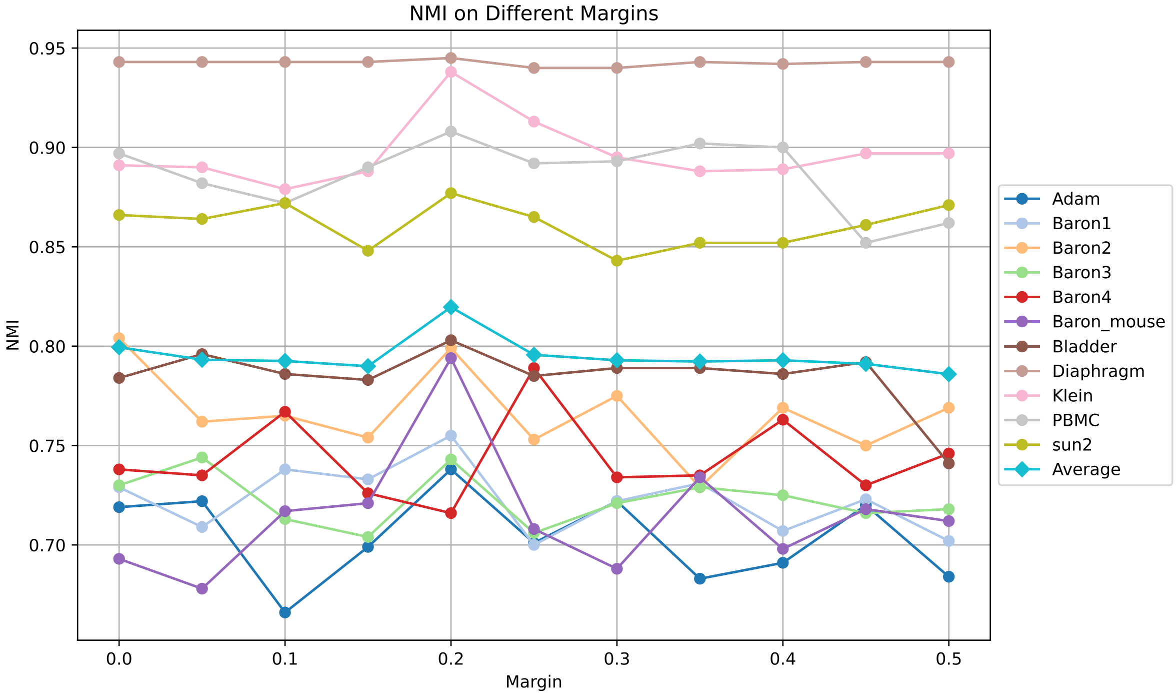

Supplementary Figure S3: The NMIs of scHNTL on different margin.

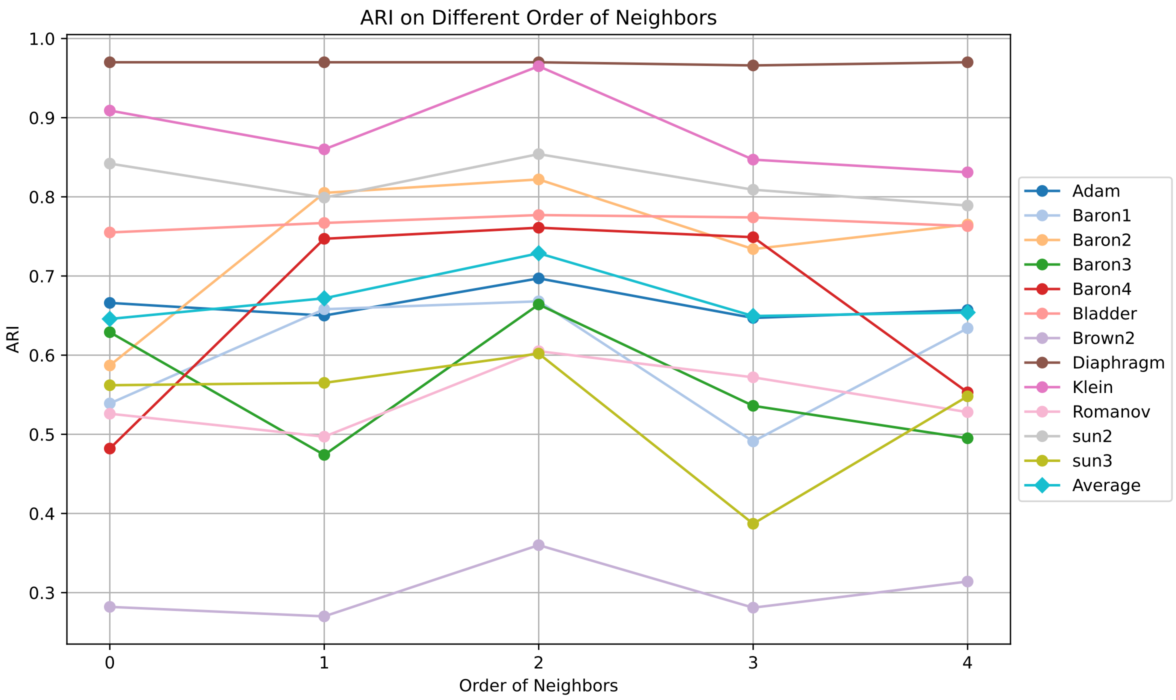

Supplementary Figure S4: The ARIs of scHNTL on different orders of neighbors.

Supplementary Figures S4 and S5 show the changes of the ARI and NMI scores when varying the order of neighbors. For most datasets, setting the largest order of neighbors to be 2 yields the best scores.

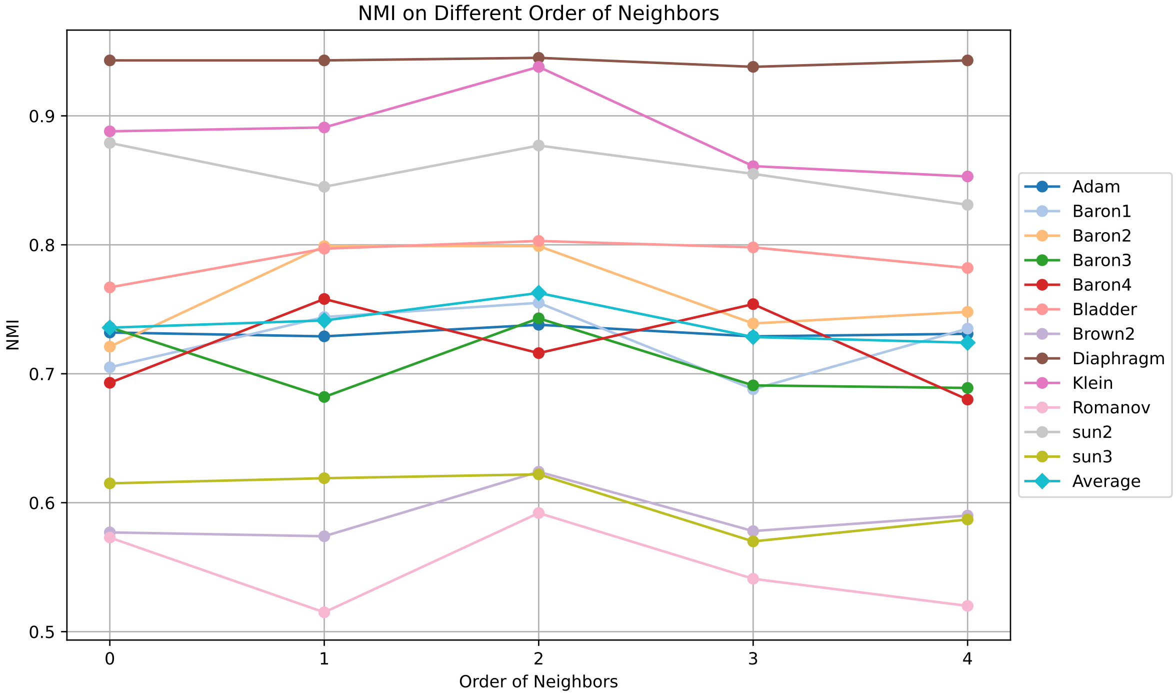

Supplementary Figure S5: The NMIs of scHNTL on different orders of neighbors.

3 Supplementary Tables

In this section, we provide the detailed values for the figures in the paper and in the previous section.

Supplementary Table S1: ARI scores of scHNTL and baseline methods.

Datasets	PCA	tsne	CIDR	SC3	DESC	scDeepCluster	scGAC	SCEA	scHNTL
Adam	0	0	0.123	0.337	<u>0.608</u>	0.58	0.601	0.463	**0.697**
Baron1	0.127	0.338	0.586	0.366	0.597	0.525	<u>0.622</u>	0.544	**0.668**
Baron2	0.274	0.351	0.302	0.407	0.642	0.629	0.746	**0.895**	<u>0.822</u>
Baron3	0.365	0.384	0.559	0.297	**0.954**	0.542	0.607	<u>0.74</u>	0.664
Baron4	0.272	0.322	0.639	0.401	0.53	0.495	0.543	<u>0.725</u>	**0.761**
Baron Mouse	0.119	0.261	0.466	0.254	0.43	<u>0.518</u>	0.405	0.431	**0.658**
Biase	0.035	0.078	**1**	0.948	<u>0.96</u>	0.948	**1**	**1**	**1**
Björklund	0.035	0.077	0.457	0.721	0.412	0.31	<u>0.785</u>	0.631	**0.872**
Brown2	0.337	0.113	0.236	0.223	0.186	0.191	0.271	0.298	**0.36**
Klein	0.298	0.544	0.677	0.803	0.789	0.762	0.835	<u>0.843</u>	**0.965**
PBMC	0.284	0.557	0.474	0.82	0.772	0.788	<u>0.854</u>	0.81	**0.939**
Qs_Diaphragm	0.008	0.026	0.925	0.801	0.723	<u>0.955</u>	**0.97**	**0.97**	**0.97**
Qx_Bladder	0.538	0.429	0.773	0.616	0.3	0.736	0.757	<u>0.775</u>	**0.777**
Romanov	0.131	0.322	0.342	0.495	0.384	0.452	0.569	<u>0.6</u>	**0.605**
Sun2	0.165	0.605	0.715	**0.899**	0.495	0.502	0.811	0.798	<u>0.854</u>
Sun3	0.112	0.195	0.528	0.426	0.244	0.331	<u>0.542</u>	<u>0.553</u>	**0.602**
Average	0.194	0.288	0.551	0.55	0.564	0.579	0.682	<u>0.692</u>	**0.763**

Methods **PCA** and **tsne** are followed with k-means. The highest scores are highlighted in bold, and the second highest scores are underlined. The average score is calculated using available results.

Supplementary Table S2: NMI scores of scHNTL and baseline methods.

Datasets	PCA	tsne	CIDR	SC3	DESC	scDeepCluster	scGAC	SCEA	scHNTL
Adam	0.003	0.003	0.051	0.207	**0.771**	0.73	0.724	0.643	<u>0.738</u>
Baron1	0.508	0.694	0.58	0.715	**0.838**	<u>0.796</u>	0.727	0.662	0.755
Baron2	0.56	0.674	0.442	0.713	0.782	0.78	0.774	**0.842**	<u>0.799</u>
Baron3	0.586	0.663	0.652	0.658	**0.918**	<u>0.763</u>	0.722	0.74	0.743
Baron4	0.55	0.629	0.66	0.719	**0.778**	<u>0.763</u>	0.694	0.736	0.716
Baron Mouse	0.474	0.603	0.511	0.658	<u>0.733</u>	0.705	0.65	0.593	**0.794**
Biase	0.087	0.137	**1**	0.929	<u>0.954</u>	0.929	**1**	**1**	**1**
Björklund	0.087	0.137	0.6	0.718	0.5	0.358	<u>0.742</u>	0.574	**0.818**
Brown2	0.353	0.345	0.487	0.535	0.529	0.483	0.577	<u>0.596</u>	**0.624**
Klein	0.415	0.573	0.657	0.858	0.78	0.806	0.856	<u>0.869</u>	**0.938**
PBMC	0.434	0.634	0.537	0.833	0.82	0.827	<u>0.855</u>	0.729	**0.908**
QS_Diaphragm	0.088	0.066	**0.969**	0.698	0.842	0.93	0.943	0.94	<u>0.945</u>
Qx_Bladder	0.543	0.537	0.734	0.517	0.572	0.781	0.777	<u>0.795</u>	**0.803**
Romanov	0.521	0.322	0.333	0.462	<u>0.589</u>	0.52	0.553	0.535	**0.592**
Sun2	0.331	0.691	0.783	**0.904**	0.764	0.72	0.861	0.842	<u>0.877</u>
Sun3	0.112	0.195	0.556	0.549	0.508	0.514	<u>0.594</u>	0.518	**0.622**
Average	0.353	0.431	0.597	0.667	0.73	0.713	<u>0.753</u>	0.726	**0.792**

Methods **PCA** and **tsne** are followed with k-means. The highest scores are highlighted in bold, and the second highest scores are underlined. The average score is calculated using available results.

Supplementary Table S3: ARI scores on different margins

margin	Adam	Baron1	Baron2	Baron3	Baron4	Baron_mouse	Bladder	Diaphragm	Klein	PBMC	sun2	Average
0	0.628	0.612	0.805	0.634	0.666	0.592	0.760	0.970	0.874	0.939	0.832	0.756
0.05	0.641	0.581	0.800	**0.684**	0.684	0.572	0.767	0.970	0.877	0.912	0.845	0.758
0.1	0.544	0.645	0.770	0.506	0.793	0.662	0.761	0.970	0.865	0.903	0.845	0.751
0.15	0.575	0.643	0.739	0.554	0.662	**0.663**	0.754	0.970	0.896	0.921	0.811	0.744
0.2	**0.697**	**0.668**	**0.822**	0.664	0.761	0.658	**0.777**	**0.970**	**0.965**	**0.939**	**0.854**	**0.798**
0.25	0.594	0.568	0.739	0.562	**0.809**	0.620	0.762	0.970	0.922	0.921	0.841	0.755
0.3	0.616	0.592	0.749	0.610	0.673	0.608	0.760	0.970	0.864	0.919	0.801	0.742
0.35	0.546	0.621	0.642	0.631	0.656	0.662	0.760	0.970	0.874	0.931	0.827	0.738
0.4	0.588	0.547	0.773	0.621	0.721	0.620	0.756	0.969	0.874	0.930	0.830	0.748
0.45	0.632	0.598	0.745	0.605	0.647	0.636	0.759	0.970	0.908	0.861	0.852	0.747
0.5	0.561	0.531	0.721	0.604	0.740	0.630	0.694	0.970	0.898	0.870	0.846	0.733

The highest scores are highlighted in bold.

Supplementary Table S4: NMI scores on different margins

margin	Adam	Baron1	Baron2	Baron3	Baron4	Baron_mouse	Bladder	Diaphragm	Klein	PBMC	sun2	Average
0	0.719	0.729	**0.804**	0.730	0.738	0.693	0.784	0.943	0.891	0.897	0.866	0.799
0.05	0.722	0.709	0.762	**0.744**	0.735	0.678	0.796	0.943	0.890	0.882	0.864	0.793
0.1	0.666	0.738	0.765	0.713	0.767	0.717	0.786	0.943	0.879	0.872	0.872	0.793
0.15	0.699	0.733	0.754	0.704	0.726	0.721	0.783	0.943	0.888	0.890	0.848	0.790
0.2	**0.738**	**0.755**	0.799	0.743	0.716	**0.794**	**0.803**	**0.945**	**0.938**	**0.908**	**0.877**	**0.820**
0.25	0.701	0.700	0.753	0.706	**0.789**	0.708	0.785	0.940	0.913	0.892	0.865	0.796
0.3	0.722	0.722	0.775	0.721	0.734	0.688	0.789	0.940	0.895	0.893	0.843	0.793
0.35	0.683	0.731	0.729	0.729	0.735	0.734	0.789	0.943	0.888	0.902	0.852	0.792
0.4	0.691	0.707	0.769	0.725	0.763	0.698	0.786	0.942	0.889	0.900	0.852	0.793
0.45	0.720	0.723	0.750	0.716	0.730	0.718	0.792	0.943	0.897	0.852	0.861	0.791
0.5	0.684	0.702	0.769	0.718	0.746	0.712	0.741	0.943	0.897	0.862	0.871	0.786

The highest scores are highlighted in bold.

Supplementary Table S5: ARI scores on different neighbor orders

orders	Adam	Baron1	Baron2	Baron3	Baron4	Bladder	Brown2	Diaphragm	Klein	Romanov	sun2	sun3	Average
0	0.666	0.539	0.587	0.629	0.482	0.755	0.282	0.970	0.909	0.526	0.842	0.562	0.646
1	0.650	0.658	0.805	0.474	0.747	0.767	0.270	0.970	0.860	0.497	0.799	0.565	0.672
2	**0.697**	**0.668**	**0.822**	**0.664**	**0.761**	**0.777**	**0.360**	**0.970**	**0.965**	**0.605**	**0.854**	**0.602**	**0.729**
3	0.647	0.491	0.734	0.536	0.749	0.774	0.281	0.966	0.847	0.572	0.809	0.387	0.649
4	0.657	0.634	0.765	0.495	0.553	0.763	0.314	0.970	0.831	0.528	0.789	0.548	0.654

The highest scores are highlighted in bold.

Supplementary Table S6: NMI scores on different neighbor orders

orders	Adam	Baron1	Baron2	Baron3	Baron4	Bladder	Brown2	Diaphragm	Klein	Romanov	sun2	sun3	Average
0	0.732	0.705	0.721	0.736	0.693	0.767	0.577	0.943	0.888	0.573	**0.879**	0.615	0.736
1	0.729	0.744	0.799	0.682	0.758	0.797	0.574	0.943	0.891	0.515	0.845	0.619	0.741
2	**0.738**	**0.755**	**0.799**	**0.743**	0.716	**0.803**	**0.624**	**0.945**	**0.938**	**0.592**	0.877	**0.622**	**0.763**
3	0.729	0.688	0.739	0.691	**0.754**	0.798	0.578	0.938	0.861	0.541	0.855	0.570	0.729
4	0.731	0.735	0.748	0.689	0.680	0.782	0.590	0.943	0.853	0.520	0.831	0.587	0.724

The highest scores are highlighted in bold.

Supplementary Table S7: ARI scores of ablation study.

Datasets	scHNTL	scHNTL-kmeans	scHNTL-1nbr
Adam	**0.697**	0.6	0.553
Baron1	**0.668**	0.654	0.624
Baron2	**0.822**	0.78	0.761
Baron3	0.664	0.468	**0.732**
Baron4	**0.761**	0.545	0.575
Baron Mouse	0.658	0.608	**0.668**
Biase	**1**	**1**	**1**
Björklund	**0.872**	0.836	0.769
Brown2	**0.36**	0.239	0.297
Klein	**0.965**	0.835	0.86
PBMC	**0.939**	0.889	0.894
QS_Diaphragm	**0.97**	**0.97**	**0.97**
Qx_Bladder	**0.777**	0.769	0.768
Romanov	**0.605**	0.532	0.533
Sun2	**0.854**	0.788	0.801
Sun3	**0.602**	0.576	0.541

Supplementary Table S8: Running time comparison (seconds).

Datasets	Number of Cells	Number of Genes	scHNTL	scGAC	SCEA
Adam	3406	17310	1260	598	565
Baron1	1918	14709	381	156	179
Baron2	1721	14881	315	136	178
Baron3	3487	15167	1285	475	455
Baron4	1289	14439	168	101	99
Baron Mouse	1883	14461	369	162	156
Biase	49	21489	37	35	19
Björklund	647	26087	98	88	72
Brown2	8605	16736	8134	7259	6656
Klein	2717	24021	760	565	464
PBMC	5356	14219	3265	2471	2332
QS_Diaphragm	869	15027	95	43	54
Qx_Bladder	2485	15357	643	229	377
Romanov	2089	18946	754	683	631
Sun2	4717	999	2374	2174	2009
Sun3	8197	1000	7624	6687	621

References

[1] Lawrence J. Hubert and Phipps Arabie. Comparing partitions. *Journal of Classification*, 2:193–218, 1985.

[2] Alexander Strehl and Joydeep Ghosh. Cluster ensembles — a knowledge reuse framework for combining multiple partitions. *Journal of Machine Learning Research*, 3:583–617, 2003.
